# A Novel Feature Extraction Method with Feature Selection to Identify Golgi-Resident Protein Types from Imbalanced Data

**DOI:** 10.3390/ijms17020218

**Published:** 2016-02-06

**Authors:** Runtao Yang, Chengjin Zhang, Rui Gao, Lina Zhang

**Affiliations:** 1 School of Control Science and Engineering, Shandong University, Jinan 250061, China; runtao-sd@163.com (R.Y.); gaorui@sdu.edu.cn (R.G.); zlnabc2010@163.com (L.Z.); 2 School of Mechanical, Electrical and Information Engineering, Shandong University at Weihai, Weihai 264209, China

**Keywords:** golgi apparatus proteins, common spatial patterns, synthetic minority over-sampling technique, recursive feature elimination, random forest

## Abstract

The Golgi Apparatus (GA) is a major collection and dispatch station for numerous proteins destined for secretion, plasma membranes and lysosomes. The dysfunction of GA proteins can result in neurodegenerative diseases. Therefore, accurate identification of protein subGolgi localizations may assist in drug development and understanding the mechanisms of the GA involved in various cellular processes. In this paper, a new computational method is proposed for identifying *cis*-Golgi proteins from *trans*-Golgi proteins. Based on the concept of Common Spatial Patterns (CSP), a novel feature extraction technique is developed to extract evolutionary information from protein sequences. To deal with the imbalanced benchmark dataset, the Synthetic Minority Over-sampling Technique (SMOTE) is adopted. A feature selection method called Random Forest-Recursive Feature Elimination (RF-RFE) is employed to search the optimal features from the CSP based features and *g*-gap dipeptide composition. Based on the optimal features, a Random Forest (RF) module is used to distinguish *cis*-Golgi proteins from *trans*-Golgi proteins. Through the jackknife cross-validation, the proposed method achieves a promising performance with a sensitivity of 0.889, a specificity of 0.880, an accuracy of 0.885, and a Matthew’s Correlation Coefficient (MCC) of 0.765, which remarkably outperforms previous methods. Moreover, when tested on a common independent dataset, our method also achieves a significantly improved performance. These results highlight the promising performance of the proposed method to identify Golgi-resident protein types. Furthermore, the CSP based feature extraction method may provide guidelines for protein function predictions.

## 1. Introduction

The Golgi Apparatus (GA), an important eukaryotic organelle involved in the metabolism of numerous proteins [1], is a major collection and dispatch station for numerous proteins destined for secretion, plasma membranes and lysosomes [2,3]. The main function of the GA is to store, package and distribute proteins [4]. In plant cells, the GA further serves as the site at which the complex polysaccharides of the cell wall are synthesized [5]. The GA is comprised of three distinct membrane-bounded cisternae located between the endoplasmic reticulum and the cell surface, including *cis*-Golgi, *media*-Golgi, and *trans*-Golgi [6]. The multiple classes of cisternae differ in structure, composition, and function. The *cis*-Golgi and *trans*-Golgi are thought to be specialised cisternae leading proteins in and out of the GA [7]. The *cis*-Golgi functions as the receiving end for the biosynthetic output from the endoplasmic reticulum [4]. The function of the *trans*-Golgi is to sort and ship proteins to their intended destinations [8]. Many different glycosyltransferases and other proteins are retained preferentially in a sub-Golgi apparatus to perform their various synthetic activities. Although the basic mechanism of the GA processing is known, how Golgi cisternae transports biosynthetic secretory cargo, and how resident Golgi proteins are localized to particular sets of cisternae, remain important and fascinating questions that await resolution [9]. Hence, to elucidate functions of the GA involved in various cellular processes, an initial but crucial step is to identify the protein composition of the subcellular compartments of the GA.

As indicated in [6], defects in Golgi apparatus can result in neurodegenerative diseases such as amyotrophic lateral sclerosis (ALS) [10], Parkinson’s disease [2], and Alzheimer’s disease (AD) [11]. The accumulation and aggregation of *β*-amyloid (A*β*) protein is one of the characteristic hallmarks of AD [12,13]. The Group 9 complexes presented in [14] have great potential as inhibitors of A*β*1-40 peptide aggregation that is linked to neurodegeneration in AD patients. Protein S-nitrosylation might represent a potentially viable therapeutic target for a wide range of neurodegenerative diseases [15]. As neuroprotective and anti-inflammatory therapies have largely proved unsatisfactory, considerable effort will be needed to make progress towards effective therapies for neurodegenerative diseases [16]. As demonstrated in [17], dysfunction of Golgi apparatus and its cisternae can give rise to muscular dystrophy, diabetes, cancers and other inheritable diseases. In addition, the GA is considered as an early target of the neurodegenerative diseases [18]. The GA is a major cargo sorting and glycosylation station [19]. Glycans have also been proved to be associated with a number of epidemic diseases such as some inherited diseases, cancers and diabetes. However, the corresponding molecular clues are only just being elucidated [17]. Accurate identification of protein subGolgi localizations could provide useful clues to clarify the contribution of GA dysfunction to diseases, which will significantly impact our ability to develop more effective therapies for diseases and spur further research into the links between glycosylation and disease pathology.

Recently, a substantial amount of machine learning methods for predicting protein subcellular locations have been developed [20,21,22]. However, few methods have been reported for predicting protein subGolgi localizations (*cis*-Golgi *vs. trans*-Golgi). In 2011, Ding *et al*. [6] employed a special mode of pseudo amino acid composition (increment of diversity) with the modified Mahalanobis discriminant to predict the types of Golgi-resident proteins. The accuracy obtained by the jackknife test was 74.7% in discriminating *cis*-Golgi proteins from *trans*-Golgi proteins. In 2013, Ding *et al*. [4] further extended their work, and presented a discriminative computational framework using *g*-gap dipeptide based protein features followed by support vector machine. The analysis of variance (ANOVA) was employed to obtain the optimal features. By the jackknife cross-validation, this method achieved an accuracy of 0.854 and an area under the receiver operating characteristic curve of 0.878. In this paper, we follow the pioneer studies aiming to further improve the prediction performance of protein subGolgi localizations (*cis*-Golgi *vs. trans*-Golgi).

The aforementioned methods were trained on relatively small datasets with no more than 150 GA proteins. Predictors trained on a dataset of limited size and coverage often fail to identify protein attributes. Recent breakthrough of proteomic techniques has resulted in a rapid growth of newly discovered protein sequences. Therefore, the benchmark datasets used in the previous methods definitely need to be updated. In addition, the dataset is highly imbalanced in [4], *i.e.*, the fraction of *trans*-Golgi proteins is relatively small compared with that of *cis*-Golgi proteins. For an imbalanced dataset, a classifier would tend to predict most of the incoming data belonging to the majority class [23]. In this study, we attempt to rebuild training sets through the SMOTE (Synthetic Minority Over-sampling Technique) to solve this imbalanced data problem.

The previous predictors to discriminate *cis*-Golgi proteins from *trans*-Golgi proteins applied only information concerning the composition of the protein chain. Evolutionary-based features have not been adequately explored, which have been successfully applied in protein attribute predictions [24,25,26]. These evolutionary-based features are extracted from the Position Specific Scoring Matrix (PSSM). Based on the concept of Common Spatial Patterns (CSP), a novel feature extraction technique is proposed in this study to extract three sets of features from PSSM-Dipeptide Composition (PSSM-DC), Bi-gram PSSM, and Evolutionary Difference-PSSM (ED-PSSM). *g*-gap dipeptide based features have attained good results in previous studies [4,6] for this task. We improve the prediction accuracy by further incorporating the three informative evolutionary patterns and *g*-gap dipeptide based features. The hybrid feature representation, containing evolutionary and sequence order information, can effectively analyze protein sequences. However, it leads to the feature vector with a high dimension. In order to reduce computation complexity and feature redundancy, the method of Random Forest-Recursive Feature Elimination (RF-RFE) is employed to find the optimal feature subset.

**Figure 1:**
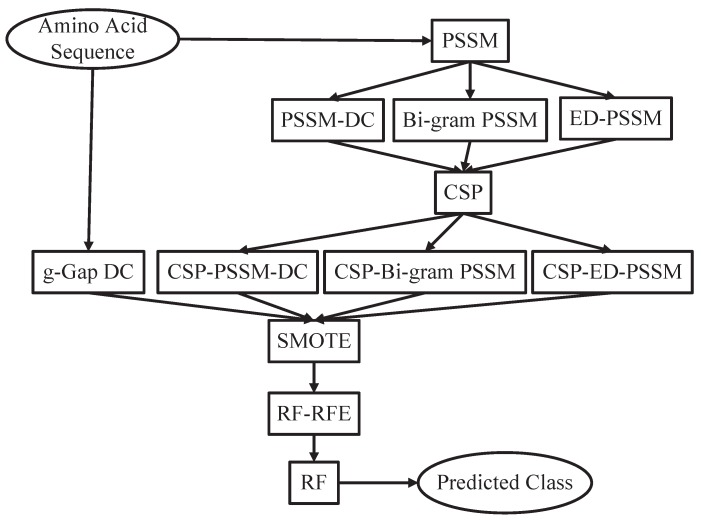
The system architecture of the proposed method. PSSM: Position Specific Scoring Matrix, DC: Dipeptide Composition, ED: Evolutionary Difference, CSP: Common Spatial Patterns, SMOTE: Synthetic Minority Over-sampling Technique, RF: Random Forest, RFE: Recursive Feature Elimination.

There are three major problems in the task of computational protein function prediction, including the construction of datasets, the extraction of protein representations, and the choice of classification algorithms [27]. The proposed prediction system is constructed based on an updated benchmark dataset. A CSP based feature extraction strategy is adopted to extract evolutionary information from protein sequences. The perdition performance of CSP based feature extraction method is comparable to that of traditional feature extraction methods. However, the feature number of the CSP based feature extraction method is only 1/20 of traditional feature extraction methods. Therefore, less computational and space cost is needed for the CSP based feature extraction method. CSP reduces computational complexity of our pipeline and effectively explore potential evolutionary information of protein sequences. In order to deal with this imbalanced data problem, we consider the SMOTE (Synthetic Minority Over-sampling Technique) to achieve balance. The Random Forest classifier is used to get an unbiased prediction. The system architecture of the proposed method is shown in Figure 1. In the 10-fold cross-validation, our method achieves an overall accuracy of 0.908 for the prediction of *cis*-Golgi proteins and an overall accuracy of 0.894 for the prediction of *trans*-Golgi proteins. To further demonstrate its advantages, the proposed method is tested on the independent dataset given by the existing method [4]. The results demonstrate that the proposed method is superior to the existing methods. Therefore, our method can be an effective predictor for large-scale determination of Golgi-resident protein types.

## 2. Results and Discussions

### 2.1. Amino Acid Composition Analysis

**Figure 2:**
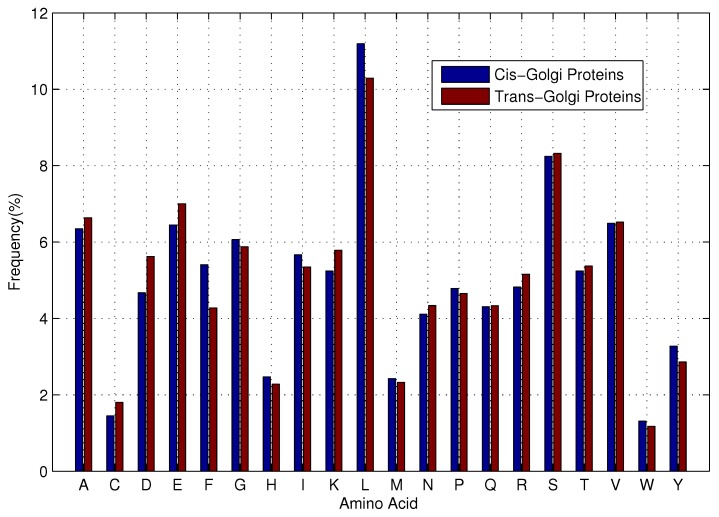
Average amino acid frequencies of *cis*-Golgi and *trans*-Golgi proteins.

To analyze the general sequence-based characteristics of *cis*-Golgi and *trans*-Golgi proteins, we calculate the average amino acid frequencies of the *cis*-Golgi and *trans*-Golgi proteins. The Figure 2 shows a bar-graph comparing the amino acid frequencies of *cis*-Golgi and *trans*-Golgi proteins.

As shown in Figure 2, *cis*-Golgi proteins share marked similar sequence composition with *trans*-Golgi proteins. Traditional computational approaches for protein function prediction have explored homology relationships using the Basic Local Alignment Search Tool (BLAST) [28]. It is a sequence similarity based method and identifies regions/segments in the query protein which are similar to the target sequences. It is clear that BLAST is inefficient in distinguishing between *cis*-Golgi and *trans*-Golgi proteins because of the high sequence composition similarity between *cis*-Golgi and *trans*-Golgi proteins. Machine learning-based algorithms are thus a good alternative for predicting Golgi-resident protein types.

### 2.2. The Impact of *g* on the Prediction Performance of *g*-Gap Dipeptide Composition

**Figure 3:**
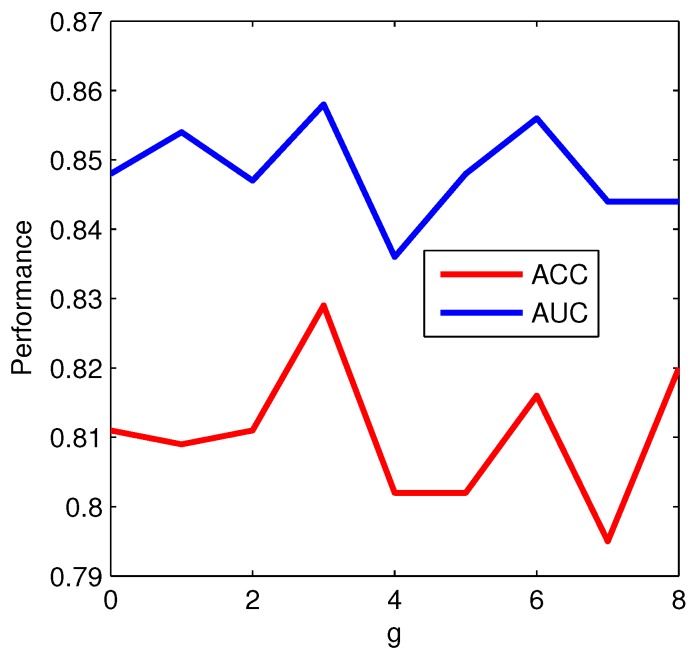
The performance of *g*-gap dipeptide composition features with various *g* values on the trainning dataset. Acc: Accuracy, AUC: Area Under the ROC Curve.

In the construction process of *g*-gap DC, the choice of the key representative parameter *g* would have a significant impact on the prediction performance. Therefore, we first investigate the impact of *g* ranging from 0 to 8 on the prediction performance. Acc and AUC are used as the main measures to determine the optimal value of *g*. The performance of *g*-gap DC transformed features for different values of *g* on the trainning dataset is shown in Figure 3. The curve demonstrates that the prediction performance is dependent on the value of *g*. With the increase of *g*, the prediction performance is not always increased. The Acc and AUC reach maximums with g=3. This result may be due to that the intrinsic properties of protein sequences is deposited in the correlation between 2 residues 3 three residue interval through the hydrogen bonding in secondary structure. Table 1 shows the detailed prediction performance of each RF model with different *g*. The model with g=3 achieves the highest Sn of 0.733, the second highest Sp of 0.926, and the highest MCC of 0.672 among various *g* values, which further validates the reliability of the performance based on g=3. In the rest of the work, 3-gap DC is considered as the baseline features. Additional features are added to the baseline features to further improve the performance.

**Table 1:** Detailed predictive results of the current method trained by *g*-gap dipeptide composition with different *g*.

*g*	Sensitivity	Specificity	Accuracy	MCC	AUC
0	0.714	0.908	0.811	0.634	0.848
1	0.724	0.894	0.809	0.627	0.854
2	0.724	0.899	0.811	0.632	0.847
3	0.733	0.926	0.829	0.672	0.858
4	0.705	0.899	0.802	0.615	0.836
5	0.700	0.903	0.802	0.616	0.848
6	0.710	0.922	0.816	0.646	0.856
7	0.700	0.889	0.795	0.601	0.844
8	0.705	0.935	0.820	0.658	0.844

### 2.3. Performance Comparison between the CSP Based Feature Extraction Method and Traditional Feature Extraction Methods from Evolutionary Information

In order to verify the effectiveness of the CSP based feature extraction method, the prediction results of the CSP based feature extraction method and traditional feature extraction methods from evolutionary information are compared. As listed in Table 2, the Acc of the CSP based feature extraction method is comparable to that of traditional feature extraction methods. The prediction accuracies of CSP-PSSM-DC, CSP-Bi-gram PSSM, and CSP-ED-PSSM are only 0.007, 0.01, and 0.009 less than those of PSSM-DC, Bi-gram PSSM, and ED-PSSM, respectively. However, the feature number of the CSP based feature extraction method is only 1/20 of traditional feature extraction methods. In real world application, the CSP based feature extraction method is preferred because compared to traditional feature extraction methods, less computational and space cost is needed. In summary, based on the computational efficiency and the prediction performance, the CSP based feature extraction method with fewer features is effective to identify Golgi-resident protein types. In the following subsection, we further improve the Acc by incorporating the CSP based feature extraction method and 3-gap DC.

**Table 2:** Prediction results of the CSP based feature extraction method and traditional feature extraction methods from evolutionary information.

Method	Sensitivity	Specificity	Accuracy	MCC	AUC	Feature Number
PSSM-DC	0.843	0.774	0.809	0.619	0.873	400
CSP-PSSM-DC	0.705	0.899	0.802	0.615	0.855	20
Bi-gram PSSM	0.710	0.922	0.816	0.646	0.909	400
CSP-Bi-gram PSSM	0.843	0.770	0.806	0.615	0.881	20
ED-PSSM	0.876	0.820	0.848	0.697	0.903	400
CSP-ED-PSSM	0.848	0.829	0.839	0.678	0.908	20

### 2.4. Predictive Capability of Combined Features

In this section, we present the performance analysis of hybrid feature sets constructed by the combination of the CSP based feature extraction method and 3-gap DC. The hybrid features are developed by simple concatenation of individual feature sets. Table 3 presents classification results of different hybrid feature sets. The first three hybrid feature sets are developed by hybridizing 3-gap DC and the three individual CSP based features. The Acc of 3-gap DC+CSP-PSSM-DC, 3-gap DC+CSP-Bi-gram PSSM, and 3-gap DC+CSP-ED-PSSM is 0.834, 0.846, and 0.859, respectively, which is marginally higher than that of CSP-PSSM-DC (0.802), CSP-Bi-gram PSSM (0.806), and CSP-ED-PSSM (0.839), respectively. 3-gap DC+CSP-ED-PSSM performs best among the first three hybrid feature sets. This probably means that CSP-ED-PSSM has a better discriminative ability for classifying *cis*-Golgi proteins from the *trans*-Golgi proteins. The feature set consisting of 3-gap DC, CSP-PSSM-DC, CSP-Bi-gram PSSM, and CSP-ED-PSSM results in maximum discrimination between *cis*-Golgi proteins and *trans*-Golgi proteins, with the Sn of 0.876, the Sp of 0.853, the Acc of 0.864, the MCC of 0.728, and the AUC of 0.912. In terms of Acc, the model trained with 3-gap DC+CSP-PSSM-DC+CSP-Bi-gram PSSM+CSP-ED-PSSM shows an improvement of 0.025–0.062 over the single feature extraction models. These results show that different features have their own merits and shortcomings, and fusion process can largely provide complementary information. It is notable from Table 3 that 3-gap DC+CSP-PSSM-DC+CSP-Bi-gram PSSM performs worse than 3-gap DC+CSP-PSSM-DC. This phenomenon indicates that not all the features are effective to improve the prediction performance. The incorporation of CSP-Bi-gram PSSM will simultaneously increase the information redundancy and deteriorate the final accuracy. To further improve the prediction performance, a proper feature selection approach should be adopted to select an optimal feature set from 3-gap DC+CSP-PSSM-DC+CSP-Bi-gram PSSM+CSP-ED-PSSM.

**Table 3:** The performance of models trained with combined features.

Training Feature	Sensitivity	Specificity	Accuracy	MCC	AUC
3-gap DC+CSP-PSSM-DC	0.853	0.816	0.834	0.669	0.887
3-gap DC+CSP-Bi-gram PSSM	0.853	0.839	0.846	0.691	0.887
3-gap DC+CSP-ED-PSSM	0.876	0.843	0.859	0.719	0.905
3-gap DC+CSP-PSSM-DC+CSP-Bi-gram PSSM	0.857	0.793	0.825	0.651	0.882
3-gap DC+CSP-PSSM-DC+CSP-ED-PSSM	0.862	0.843	0.853	0.705	0.899
3-gap DC+CSP-Bi-gram PSSM+CSP-ED-PSSM	0.843	0.839	0.841	0.682	0.894
3-gap DC+CSP-PSSM-DC+CSP-Bi-gram PSSM+CSP-ED-PSSM	0.876	0.853	0.864	0.728	0.912

### 2.5. Performance of the Current Method with or without SMOTE

In order to investigate the effectiveness of SMOTE in solving the imbalanced dataset problem, the models trained with or without SMOTE are constructed, respectively. Prediction results of the models with or without SMOTE are shown in Table 4. After directly performing the 10-fold cross-validation on the training dataset without SMOTE, the Acc and Sp are 0.730 and 0.949. However, the Sn is as low as 0.184 due to the imbalanced data size. The SMOTE based model achieves a Sn of 0.876, Acc of 0.864, and MCC of 0.728, far better than the training results without SMOTE. Although the Sp of the SMOTE based model is lower than that of model without SMOTE, the model with SMOTE achieves a more balanced Sn (0.876) and Sp (0.853).

**Table 4:** Prediction results with and without SMOTE.

Method	Sensitivity	Specificity	Accuracy	MCC
Without SMOTE	0.184	0.949	0.730	0.048
With SMOTE	0.876	0.853	0.864	0.728

**Figure 4:**
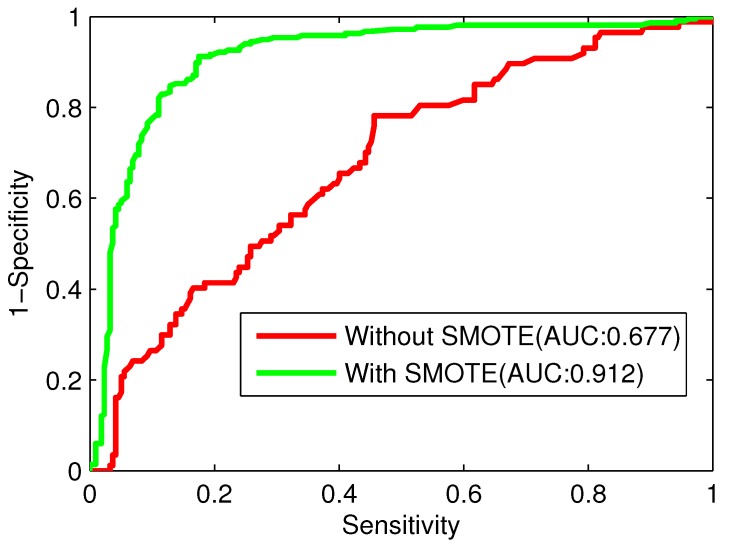
ROC (Receiver-Operating Characteristic) curves with and without SMOTE.

On the other hand, we create ROC curves with and without SMOTE to further demonstrate the effectiveness of SMOTE in solving the imbalanced dataset problem. As shown in Figure 4, the ROC curve with SMOTE is above the ROC curve without SMOTE. The AUC criterion is dramatically improved from 0.677 to 0.912 by introducing SMOTE. These results provide strong evidence that SMOTE is a very promising way for selecting more informative and representative data subset to deal with the imbalanced data problem.

### 2.6. Feature Selection Results

**Figure 5:**
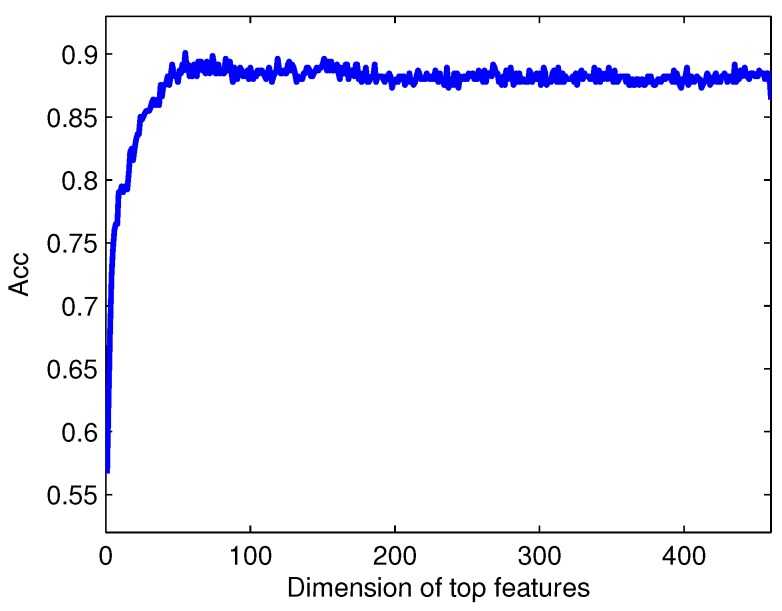
The Acc value against the dimension of top features. The maximum Acc value is 0.901 when the first 55 features in the ranked feature list are selected.

Generally, redundant and irrelevant features exist in the original feature set, which can result in over-fitting, information redundancy and dimension disaster [54]. Feature selection is another critical step in classification. By decreasing the model’s complexity, the selection of the optimal features can reduce the risk of over-fitting and enhance the efficiency. We run the RF-RFE algorithm to get a rank list of all features by removing only one feature with the lowest influence on the prediction performance each time. Within the list (see Table S2), a feature with a smaller index indicates that it is a more important feature for Golgi-resident protein type prediction. Four-hundred sixty individual classifiers are built by removing features one by one from the bottom of the feature list to the top. The detailed prediction results against different numbers of features can be found in Table S3. The Acc values of predictors against different numbers of features are shown in Figure 5. The peak of the curve appears with the Acc of 0.901 when the top 55 features (approximate 12% of the original 460 features) are selected, which demonstrate that many features in the original feature set are redundant and irrelevant. These selected features are considered as the optimal feature set used in our final prediction model. For these 55 features, please refer to the top 55 features listed in the Table S2.

Table 5 shows the 10 fold cross-validation prediction results using 3-gap DC+CSP-PSSM-DC+CSP-Bi-gram PSSM+CSP-ED-PSSM with feature selection (RF-RFE) and without feature selection. The performance of the predictor using the optimal feature set is better than that of the predictor using all 460 features, with the results for Sn, Sp, Acc, and MCC increasing from 0.876, 0.853, 0.864, and 0.728 to 0.908, 0.894, 0.901, and 0.802, respectively.

**Table 5:** Prediction results for Golgi-resident protein types using 3-gap DC+CSP-PSSM-DC+CSP-Bi-gram PSSM+CSP-ED-PSSM with and without feature selection.

Method	Sensitivity	Specificity	Accuracy	MCC	Feature Number
without feature selection	0.876	0.853	0.864	0.728	460
With feature selection	0.908	0.894	0.901	0.802	55

**Figure 6:**
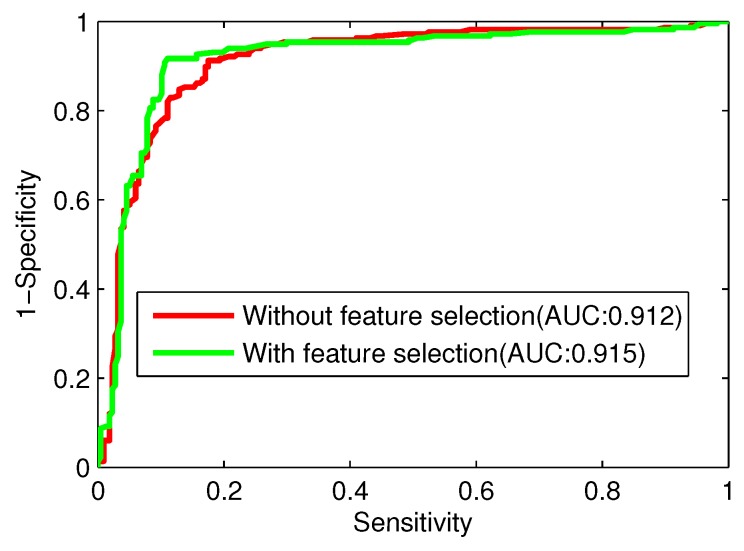
ROC (Receiver-Operating Characteristic) curves with and without feature selection.

To further demonstrate the prediction power of the RF-RFE algorithm, ROC curves with and without feature selection are illustrated in Figure 6. The AUC with feature selection is 0.915 for the trainning dataset, which is higher than that without feature selection. Our results demonstrate that the proposed feature selection technique (RF-RFE) can effectively improve the prediction performance.

### 2.7. Performance Comparison with the Existing Methods

In order to demonstrate the reliability and efficiency of the proposed method, we make comparisons with previously published methods by the jackknife cross validation. Detailed comparison results are summarized in Table 6, where better results are highlighted in bold. From Table 6, the prediction results of our method are much better than those of other methods. The Acc and MCC obtained by our model are 0.885 and 0.765, respectively, which are significantly higher than those of available methods. In terms of Sn and Sp, our method achieves 0.889 and 0.880, which suggests that our method has a relatively balanced performance in positive and negative datasets. In contrast, there is a great divergence between Sn and Sp in [4] and [6]. Although the Sp of the method given in [4] is 0.025 higher than that of our method, the Sn, Acc and MCC are 0.151, 0.031, and 0.113 lower than that of our method, respectively. These results indicate that our proposed method is able to significantly enhance the prediction accuracy compared to the previous studies and at the same time reduce the number of features used for this task remarkablely.

**Table 6:** Performance comparisons with the existing methods on the training dataset by the jackknife cross validation, where better results are highlighted in bold.

Reference	Sensitivity	Specificity	Accuracy	MCC	Feature Number
[6]	0.696	0.796	0.747	0.517	400
[4]	0.738	**0.905**	0.854	0.652	83
This study	**0.889**	0.880	**0.885**	**0.765**	**55**

To further evaluate the prediction performance of the current method objectively, it is necessary to compare it with other existing methods on the independent testing dataset. As the web-server of the computational predictor provided by [6] is unavailable, the comparison is carried out between our method and the method proposed in [4] on the independent testing dataset introduced by [4]. The detailed comparison results are listed in Table 7. Our method yields the Sn, Sp, Acc, and MCC values of 0.923, 0.941, 0.938, and 0.821, which are 0.231, 0.039, 0.079, and 0.243 higher than those obtained by [4]. These results highlight the promising performance of the proposed method to tackle the Golgi-resident protein type prediction problem.

**Table 7:** Performance comparison with the existing methods on the independent testing dataset.

Reference	Sensitivity	Specificity	Accuracy	MCC	Feature Number
[4]	0.692	0.902	0.859	0.578	83
This study	0.923	0.941	0.938	0.821	55

The outstanding performance of the current method may be attributed to four aspects. (i) The perdition performance of CSP based feature extraction method is comparable to that of traditional feature extraction methods. However, the feature number of the CSP based feature extraction method is only 1/20 of traditional feature extraction methods. Therefore, less computational and space cost is needed for the CSP based feature extraction method. CSP reduces computational complexity of our pipeline and effectively explore potential evolutionary information of protein sequences; (ii) A combination of feature extraction methods integrates complementary information of protein sequences; (iii) To make the number of *cis*-Golgi samples be equal to the number of *trans*-Golgi samples, new *cis*-Golgi samples in the feature spaces are generated via SMOTE algorithm. The model with SMOTE achieves a more balanced *Sn* (0.876) and *S*p (0.853). SMOTE is an effective method for selecting more informative and representative data subset to deal with the imbalanced data problem that exists in our pipeline; (iv) A feature selection method called RF-RFE (Random Forest-Recursive Feature Elimination) is employed to pick out high discriminative features. Based on RF-RFE, 55 features are considered as the optimal feature set used in our final prediction model. The performance of the predictor using the optimal feature set is better than that of the predictor using all 460 features, with the results for Sn, Sp, Acc, and MCC increasing from 0.876, 0.853, 0.864, and 0.728 to 0.908, 0.894, 0.901, and 0.802, respectively. These results demonstrate that the proposed feature selection technique (RF-RFE) can effectively improve the prediction performance.

## 3. Materials and Methods

### 3.1. Datasets

To update the training datasets introduced by Ding *et al*. [4,6], the Golgi-resident proteins applied in this study are collected from the latest Universal Protein KnowledgeBase (UniProtKB), which provides the scientific community with a comprehensive, high quality and freely accessible resource of protein sequences [29]. To search the *cis*-Golgi proteins or *trans*-Golgi proteins, respectively, we use the keyword of subcellular locations (“*cis*-Golgi” or “*trans*-Golgi”) and add restrictions, that is “fragment: not”, “containing nonstandard letters: yes”, and “reviewed: yes”. These restrictions are applied to reduce the redundant, incomplete, and incorrect information. Sequences included in the independent testing dataset given in [4] are excluded because they may lead to overfitting problem. To avoid homology bias, we remove the redundant sequences using CD-HIT with a 40% identity cutoff [30]. As a result, the training dataset consists of 87 *cis*-Golgi proteins and 217 *trans*-Golgi proteins.

In order to facilitate comparison with previous studies, a dataset composed of 13 *cis*-Golgi proteins and 51 *trans*-Golgi proteins, introduced by Ding *et al*. [4], is employed to construct the independent testing dataset. The benchmark dataset adopted in this study is available in Table S1.

Predicting Golgi-resident protein types is formulated as a two class classification problem, where *cis*-Golgi proteins belong to the positive class and *trans*-Golgi proteins to the negative class.

### 3.2. Feature Extraction

For developing a powerful predictor, it is significant to convert the input protein sequence into a set of numerical features that could really reflect the intrinsic correlation with the desired target [31]. Commonly, the combination of various features from different sources can take full advantage of the supplementary information from protein samples [32,33]. In this study, dipeptide composition and evolutionary information are combined to transform the protein sequences into feature vectors. Three traditional feature extraction methods namely, PSSM-DC, Bi-gram PSSM, and ED-PSSM, are adopted to extract evolutionary information from the PSSM. Based on the concept of CSP, a novel feature extraction technique is proposed to extract features from PSSM-DC, Bi-gram PSSM, and ED-PSSM, respectively. More details about these feature extraction methods will be explained in the following subsections.

#### 3.2.1. *g*-Gap Dipeptide Composition

The diversity and specificity of protein structures and functions are largely attributed to amino acid compositions [34]. Adjoining dipeptide composition represents the occurrence frequency of each two adjacent amino acid residues. Compared to the amino acid composition, the adjoining dipeptide composition encapsulates both the fraction information of amino acids and the local order information of protein sequences, which has been used for protein attribute predictions [35,36]. Without considering the intrinsic properties deposited in the correlations between spatially close amino acid residues [37,38,39], the adjoining dipeptide composition can only depict the correlation between two adjoining amino acids. Thus, the *g*-gap Dipeptide Composition proposed in [4] is employed in this study to search for the important correlation between two residues.

For a protein sequence *P* with *L* residues, the *g*-gap dipeptide composition can be expressed as follows.

(1) $$F^{g}={\left\{f_{1}^{g},f_{2}^{g},f_{i}^{g},\cdots ,f_{400}^{g}\right\}}^{T}$$

where the symbol *T* denotes the transpose of the vector. *g* is the number of intervening residue. $f_{i}^{g}$ denotes the frequency of the *i*th *g*-gap dipeptide and is defined as

(2) $$f_{i}^{g}=\frac{n_{i}^{g}}{L-g-1}$$

where $n_{i}^{g}$ denotes the number of the *i*th *g*-gap dipeptide.

#### 3.2.2. Traditional Feature Extraction Methods from Evolutionary Information

As one of the most important aspects in biological sequence analysis, evolutionary conservation, reflects important biological functions [40]. Conserved sequences are similar or identical sequences that still share many common features during the evolution process [41]. A functionally important region is always conservative in the evolutionary process [42]. Exploiting the detailed conservation pattern of residues will largely facilitate the prediction of protein functions [43]. PSSM has been widely used to transform the variable lengths of protein sequences into fixed-length feature vectors while keeping considerable evolutionary information [44,45,46].

The PSI-BLAST (Position-Specific Iterative Basic Local Alignment Search Tool) [28] is used to generate PSSM by searching homogenous sequences for each query protein through three iterations with 0.001 as the E-value cutoff. The search is performed against the Swiss-Prot database. PSSM profile for each query protein can be expressed as

(3) $$P_{\mathrm{PSSM}}=\left[\begin{array}{cccccc} E_{1\to 1} & E_{1\to 2} & \cdots & E_{1\to j} & \cdots & E_{1\to 20}\\ E_{2\to 1} & E_{2\to 2} & \cdots & E_{2\to j} & \cdots & E_{2\to 20}\\ \vdots & \vdots & \cdots & \vdots & \cdots & \vdots \\ E_{i\to 1} & E_{i\to 2} & \cdots & E_{i\to j} & \cdots & E_{i\to 20}\\ \vdots & \vdots & \cdots & \vdots & \cdots & \vdots \\ E_{L\to 1} & E_{L\to 2} & \cdots & E_{L\to j} & \cdots & E_{L\to 20} \end{array}\right]$$

where *L* is the length of the query sequence and the values of j=1,2,⋯,20 represent the 20 native amino acids according to their alphabetical order. $E_{i\to j}$ can be interpreted as the relative probability of *j*th amino acid at the *i*th location of the query sequence during the evolution process. Large positive scores often indicate critical functional residues. In this study, three traditional feature extraction methods namely, PSSM-DC, Bi-gram PSSM, and ED-PSSM, are adopted to extract evolutionary information from the PSSM.

**(1) PSSM-Dipeptide Composition**

Previous works have exhibited the ability of PSSM-dipeptide composition (PSSM-DC) in the protein function predictions [47,48,49]. PSSM-DC transforms L×20 PSSM into 20×20 PSSM as formulated by

(4) $$\mathrm{PSSM}-\mathrm{DC}=\left[\begin{array}{cccc} \sum E_{A\to A} & \sum E_{A\to R} & \cdots & \sum E_{A\to V}\\ \sum E_{R\to A} & \sum E_{R\to R} & \cdots & \sum E_{R\to V}\\ \vdots & \vdots & \vdots & \vdots \\ \sum E_{V\to A} & \sum E_{V\to R} & \cdots & \sum E_{V\to V} \end{array}\right]$$

where $\sum E_{i\to j}$ denotes the sum of amino acid type *i* being changed to amino acid type *j* in Equation (3), followed by division of each element by the length of the sequence.

**(2) Bi-Gram PSSM**

Bi-gram features directly extracted from PSSM have been adopted in recent studies [50,51] to address the shortcoming that the computed bi-gram feature vector from the original protein sequence is very sparse. Bi-gram PSSM computes the frequency of occurrence of transition from *m*th amino acid to *n*th amino acid as follows:

(5) $$B_{m,n}=\sum_{i=1}^{L-1}E_{i\to m}E_{i+1\to n},\;m,n=1,2,\cdots ,20$$

The values of (m,n=1,2,⋯,20) denote the 20 native amino acids according to their alphabetical order. Equation (6) gives 400 frequencies of occurrences, which can be formulated as

(6) $$B={\left[B_{1,1},B_{1,2},\cdots ,B_{1,20};B_{2,1},\cdots ,B_{2,20};\cdots ;B_{20,1},\cdots ,B_{20,20}\right]}^{T}$$

where *T* denotes the transpose of the vector.

**(3) Evolutionary Difference-PSSM**

Evolutionary Difference-PSSM is proposed to represent mutation difference between adjacent residues. A given protein can be expressed as a 20×20 matrix ED-PSSM denoted by

(7) $$ED-PSSM=(e_{1},e_{2},\cdots ,e_{m},\cdots ,e_{20}),m=1,2,\cdots ,20$$

where

(8) $$e_{m}={\left(e_{1,m},e_{2,m},\cdots ,e_{n,m},\cdots ,e_{n,20}\right)}^{T},n=1,2,\cdots ,20$$

(9) $$e_{m,n}=\sum_{i=2}^{L-1}\frac{{\left(E_{i-1\to m}-E_{i+1\to n}\right)}^{2}}{L-2}$$

#### 3.2.3. Common Spatial Patterns Based Feature Extraction from Evolutionary Information

The method of common spatial patterns (CSP) has been applied successfully to extract discriminatory information from two populations of single-trial electroencephalograph [52]. In this study, we apply the concept of CSP to extract features from PSSM-DC, Bi-gram PSSM, and ED-PSSM, respectively.

Through PSSM-DC, Bi-gram PSSM, or ED-PSSM, the protein sequence is represented as a 20×20 matrix *E*. The normalized spatial covariance of the protein sequence can be obtained from

(10) $$R=\frac{EE^{\prime }}{trace(EE^{\prime })}$$

where ${}^{\prime }$ denotes the transpose operator and trace(x) is the sum of the diagonal elements of *x*. The composite spatial covariance is given as

(11) $$R_{c}={\bar{R}}_{1}+{\bar{R}}_{2}$$

where the spatial covariance ${\bar{R}}_{1}$ is calculated by averaging over the *cis*-Golgi protein sequences and the spatial covariance ${\bar{R}}_{2}$ is calculated by averaging over the *trans*-Golgi protein sequences. $R_{c}$ can be factored as $R_{c}=U_{c}\lambda_{c}U_{c}^{\prime }$, where $U_{c}$ is the matrix of eigenvectors and $\lambda_{c}$ is the diagonal matrix of eigenvalues.

The whitening transformation $P=\sqrt{\lambda_{c}^{-1}}U_{c}^{\prime }$ equalizes the variances in the space spanned by $U_{c}$, *i.e.*, all eigenvalues of $PR_{C}P^{\prime }$ are equal to one. If ${\bar{R}}_{1}$ and ${\bar{R}}_{2}$ are transformed as

(12) $$S_{1}=P{\bar{R}}_{1}P^{\prime },S_{2}=P{\bar{R}}_{2}P^{\prime }$$

then $S_{1}$ and $S_{2}$ share common eigenvectors, *i.e.*, if $S_{1}=B\lambda_{1}B^{\prime }$, then $S_{2}=B\lambda_{2}B^{\prime }$ and $\lambda_{1}+\lambda_{2}=I$, where *I* is the identity matrix. This property indicates that for a same eigenvector, the corresponding eigenvalue for $S_{1}$ is the largest (smallest) while the corresponding eigenvalue for $S_{2}$ is the smallest (largest). Therefore, the eigenvectors is suitable to extract features for classification.

With the projection matrix $W={\left(B^{\prime }P\right)}^{\prime }$, the mapping of a protein sequence is given as

(13) Z=WE

The feature vector $F=\{f_{1},f_{2},\cdots ,f_{20}\}$ used for classification is obtained by

(14) $$f_{j}=\log\left(\frac{var(Z_{j})}{\sum_{i=1}^{20}var\left(Z_{i}\right)}\right),j=1,2,\cdots ,20$$

where the subscript of *Z* denotes the column number of matrix *Z*.

Based on the method of CSP, the features extracted from PSSM-DC, Bi-gram PSSM, and ED-PSSM are denoted as CSP-PSSM-DC, CSP-Bi-gram PSSM, and CSP-ED-PSSM, respectively.

### 3.3. Synthetic Minority Over-Sampling Technique

As described in the “Datasets” section, the number of *cis*-Golgi proteins is much smaller than that of *trans*-Golgi proteins. This leads to the imbalanced data classification problem. In order to deal with this imbalanced data problem, we consider the SMOTE (Synthetic Minority Over-sampling Technique) to achieve balance. To over-sampling the minority class, SMOTE selects a minority class sample and creates novel synthetic samples along the line segment joining some or all *k* nearest neighbors belonging to that class [53]. In this paper, to make the number of *cis*-Golgi samples be equal to the number of *trans*-Golgi samples, new *cis*-Golgi samples in the feature spaces are generated via SMOTE algorithm. Subsequently, this balanced dataset, having an equal number of *cis*-Golgi and *trans*-Golgi samples, is used for training the predictor.

### 3.4. Feature Selection

The generated features by the above-mentioned feature extraction methods may be irrelevant to the prediction of golgi-resident protein types, which can result in over-fitting, information redundancy and dimension disaster [54]. To select high discriminative features and reduce computational complexity, the feature selection procedure is always indispensable in protein function predictions based on machine learning methods [55,56].

In this study, a feature selection method called RF-RFE (Random Forest-Recursive Feature Elimination) is employed to pick out high discriminative features. The RF-RFE algorithm starts with all input features and removes one feature with the lowest influence on the performance of the RF model from the feature set at each iteration. As there are 460 features in the original feature set, 460 iterations are carried out to extract the optimal features. The parameter “Accuracy” is used to evaluate the influence on the performance of the RF model. The first removed feature is the most unimportant feature; the second removed feature is the second most unimportant feature;⋯; the last removed feature is the most important feature. We run the RF-RFE algorithm to get a rank list according to the feature importance. A new feature set is constructed when another feature has been removed. The feature set that yields the highest cross-validation accuracy among all iterations is selected as the optimal feature set.

### 3.5. Classifier

The random forest (RF) algorithm, developed by Breiman [57], has been successfully applied in the field of protein function predictions [58,59]. The ensemble of decision trees generated by RF gives a good tolerance for the noisy data [57]. The decision trees are trained on different bootstrap samples from the training data. Each tree is fully grown without pruning. At each node, *m* features are selected randomly out of all features and the most optimized split on these *m* features is employed to split the node. For a new object, each decision tree gives a classification result. Based on the classification results of decision trees, RF assigns the new object a class label through majority voting.

The RF algorithm is implemented by the WEKA software package (Waikato Environment for Knowledge Analysis) [60], where default parameters are employed.

### 3.6. Performance Measures

Three methods, *i.e.*, the jackknife test, sub-sampling test, and independent dataset test are often used for examining the quality of a statistical prediction method [61]. The outcome obtained by the jackknife test is always unique for a given benchmark dataset [24]. However, to reduce the computational time, a 10-fold cross-validation test is adopted in this study. The whole dataset is randomly separated into ten parts. Each time, one part is for testing and the other nine parts form the training dataset. This process is repeated ten times to test each part.

Sensitivity (Sn), specificity (Sp), accuracy (Acc), and Matthew’s Correlation Coefficient (MCC) are employed to evaluate the performance of the prediction system. These measurements are defined as follows.

(15) $$S_{n}=\frac{TP}{TP+FN}$$

(16) $$S_{p}=\frac{TN}{TN+FP}$$

(17) $$Acc=\frac{TP+TN}{TP+FP+TN+FN}$$

(18) $$MCC=\frac{TP\times TN-FP\times FN}{\sqrt{\left(TP+FN)(TP+FP)(TN+FP)(TN+FN\right)}}$$

where TP, FP, TN and FN represent true positive (correctly predicted *cis*-Golgi proteins), false positive (*trans*-Golgi proteins incorrectly predicted as *cis*-Golgi proteins), true negative (correctly predicted *trans*-Golgi proteins) and false negative (*cis*-Golgi proteins incorrectly predicted as *trans*-Golgi proteins), respectively.

Sn measures the proportion of the known *cis*-Golgi proteins that are correctly predicted as *cis*-Golgi proteins and Sp measures the proportion of the known *trans*-Golgi proteins that are correctly predicted as *trans*-Golgi proteins. Acc denotes the percent of correct prediction in both the positive and negative sets. MCC is a weighted measure, and has been increasingly used for measuring the predictive capability of classifiers, which reflects both the sensitivity and specificity of the prediction algorithm.

We also use the receiver-operating characteristic (ROC) curve to further evaluate the performance of the proposed method. The ROC curve, one of the most reliable approaches in evaluating performance of classifiers [62], is obtained by plotting sensitivity on the *y*-axis against 1-specificity on the *x*-axis. The area under the ROC curve (AUC) is regard as a reliable measure for the performance measurement.

## 4. Conclusions

In this paper, a novel feature extraction method based on CSP has been presented to extract evolutionary information from protein sequences. The prediction performance of the CSP based feature extraction method is comparable to that of traditional feature extraction methods, but less computational and space cost is needed. We present the performance analysis on hybrid feature sets constructed by the combination of the CSP based feature extraction method and 3-gap DC. The feature set consisting of 3-gap DC, CSP-PSSM-DC, CSP-Bi-gram PSSM, and CSP-ED-PSSM results in maximum discrimination between *cis*-Golgi proteins and *trans*-Golgi proteins. These results show that different features have their own merits and shortcomings, and fusion process can largely provide complementary information. Then, the effectiveness of SMOTE in solving the imbalanced dataset problem has been investigated. The prediction performance of the SMOTE based model is far better than the training results without SMOTE. By means of the RF-RFE algorithm, 55 optimal features are selected from 3-gap DC+CSP-PSSM-DC+CSP-Bi-gram PSSM+CSP-ED-PSSM. The performance of the predictor using the optimal feature set is better than that of the predictor using all 460 features. When compared with previously published methods by jackknife cross validation, the proposed method remarkably outperforms previous methods with a Sn of 0.889, a Sp of 0.880, an Acc of 0.885, and a MCC of 0.765. Moreover, when tested on a common independent dataset, our method also achieves a significantly improved performance. These results indicate that our method has a fairly good capability to distinguish *cis*-Golgi proteins from *trans*-Golgi proteins.

## Supplementary Materials

Supplementary materials can be found at http://www.mdpi.com/1422-0067/17/2/218/s1. Table S1: The training dataset and the independent testing dataset (.xls). The training dataset consists of 87 *cis*-Golgi proteins and 217 *trans*-Golgi proteins while the independent testing dataset consists of 13 *cis*-Golgi proteins and 51 *trans*-Golgi proteins. Table S2: The ranked feature list given by the RF-RFE algorithm (.xls). Within the list, a feature with a smaller index indicates that it is more important for identifying Golgi-resident protein types. Table S3. The detailed prediction results against different numbers of features (.xls).

## References

[1] Hu Z., Zeng L., Xie L., Lu W., Zhang J., Li T., Wang X. (2007). Morphological alteration of golgi apparatus and subcellular compartmentalization of TGF-*β* 1 in Golgi apparatus in gerbils following transient forebrain ischemia. Neurochem. Res..

[2] Fujita Y., Ohama E., Takatama M., Al-Sarraj S., Okamoto K. (2006). Fragmentation of Golgi apparatus of nigral neurons with *α*-synuclein-positive inclusions in patients with Parkinson’s disease. Acta Neuropathol..

[3] Jiao Y.S., Du P.F. (2015). Predicting Golgi-resident protein types using pseudo amino acid compositions: Approaches with positional specific physicochemical properties. J. Theor. Biol..

[4] Ding H., Guo S.H., Deng E.Z., Yuan L.F., Guo F.B., Huang J., Rao N., Chen W., Lin H. (2013). Prediction of Golgi-resident protein types by using feature selection technique. Chemom. Intell. Lab. Syst..

[5] Cooper G.M., Hausman R.E. (2006). The Cell: A Molecular Approach.

[6] Ding H., Liu L., Guo F.B., Huang J., Lin H. (2011). Identify Golgi protein types with modified mahalanobis discriminant algorithm and pseudo amino acid composition. Protein Pept. Lett..

[7] Pfeffer S.R. (2001). Constructing a Golgi complex. J. Cell Biol..

[8] Pavelk M., Mironov A.A. (2008). The Golgi Apparatus: State of the Art 110 yEars after Camillo Golgi’s Discovery.

[9] Day K.J., Staehelin L.A., Glick B.S. (2013). A three-stage model of Golgi structure and function. Histochem. Cell Biol..

[10] Fujita Y., Okamoto K. (2005). Golgi apparatus of the motor neurons in patients with amyotrophic lateral sclerosis and in mice models of amyotrophic lateral sclerosis. Neuropathology.

[11] Gonatas N.K., Gonatas J.O., Stieber A. (1998). The involvement of the Golgi apparatus in the pathogenesis of amyotrophic lateral sclerosis, Alzheimer’s disease, and ricin intoxication. Histochem. Cell Biol..

[12] Leung C.H., Zhong H.J., Chan D.S.H., Ma D.L. (2013). Bioactive iridium and rhodium complexes as therapeutic agents. Coord. Chem. Rev..

[13] Ma D.L., He H.Z., Leung K.H., Chan D.S., Leung C.H. (2013). Bioactive luminescent transition-metal complexes for biomedical applications. Angew. Chem. Int. Ed. Engl..

[14] Man B.Y.W., Chan H.M., Leung C.H., Chan D.S.H., Bai L.P., Jiang Z.H., Li H.W., Ma D.L. (2011). Group 9 metal-based inhibitors of *β*-amyloid (1-C40) fibrillation as potential therapeutic agents for Alzheimer’s disease. R. Soc. Chem..

[15] Nakamura T., Lipton S.A. (2016). Protein S-nitrosylation as a therapeutic target for neurodegenerative diseases. Trends Pharmacol. Sci..

[16] Brettschneider J., Del Tredici K., Lee V.M., Trojanowski J.Q. (2015). Spreading of pathology in neurodegenerative diseases: A focus on human studies. Nat. Rev. Neurosci..

[17] Ungar D. (2009). Golgi linked protein glycosylation and associated diseases. Semin. Cell Dev. Biol..

[18] Fujita Y., Okamoto K. (2005). Golgi apparatus of the motor neurons in patients with amyotrophic lateral sclerosis and in mice models of amyotrophic lateral sclerosis. Neuropathology.

[19] Nakano A., Luini A. (2010). Passage through the Golgi. Curr. Opin. Cell Biol..

[20] Yu D., Wu X., Shen H., Yang J., Tang Z., Qi Y., Yang J. (2012). Enhancing membrane protein subcellular localization prediction by parallel fusion of multi-view features. IEEE Trans. Nanobiosci..

[21] Fan G.L., Li Q.Z. (2012). Predicting protein submitochondria locations by combining different descriptors into the general form of Chou’s pseudo amino acid composition. Amino Acids.

[22] Huang C., Yuan J.Q. (2013). Predicting protein subchloroplast locations with both single and multiple sites via three different modes of Chou’s pseudo amino acid compositions. J. Theor. Biol..

[23] Asadabadi E.B., Abdolmaleki P. (2013). Predictions of protein-protein interfaces within membrane protein complexes. Avicenna J. Med. Biotechnol..

[24] Zhang S.L., Ye F., Yuan X.G. (2012). Using principal component analysis and support vector machine to predict protein structural class for lowsimilarity sequences via PSSM. J. Biomol. Struct. Dyn..

[25] Zou L.Y., Nan C.H., Hu F.Q. (2013). Accurate prediction of bacterial type IV secreted effectors using amino acid composition and PSSM profiles. Bioinformatics.

[26] Paliwal K.K., Sharma A., Lyons J., Dehzangi A. (2014). A tri-gram based feature extraction technique using linear probabilities of position specific scoring matrix for protein fold recognition. IEEE Trans. Nanobiosci..

[27] Bernardes J.S., Pedreira C.E. (2013). A review of protein function prediction under machine learning perspective. Recent. Pat. Biotechnol..

[28] Altschul S.F., Madden T.L., Schaffer A.A., Zhang J., Zhang Z., Miller W., Lipman D.J. (1997). Gapped BLAST and PSI-BLAST: A new generation of protein database search programs. Nucleic Acids Res..

[29] Magrane M., Consortium U. (2011). UniProt knowledgebase: A hub of integrated protein data. Database.

[30] Huang Y., Niu B., Gao Y., Fu L., Li W. (2010). CD-HIT Suite: A web server for clustering and comparing biological sequences. Bioinformatics.

[31] Chou K.C. (2011). Some remarks on protein attribute prediction and pseudo amino acid composition. J. Theor. Biol..

[32] Han G.S., Yu Z.G., Anh V., Krishnajith A.P., Tian Y.C. (2013). An ensemble method for predicting subnuclear localizations from primary protein structures. PLoS ONE.

[33] Shi S.P., Qiu J.D., Sun X.Y., Suo S.B., Huang S.Y., Liang R.P. (2012). A method to distinguish between lysine acetylation and lysine methylation from protein sequences. J. Theor. Biol..

[34] Zhang C.T., Chou K.C. (1992). An optimization approach to predicting protein structural class from amino acid composition. Protein Sci..

[35] Kaundal R., Saini R., Zhao P.X. (2010). Combining Machine Learning and Homology-Based Approaches to Accurately Predict Subcellular Localization in arabidopsis. Plant Physiol..

[36] Lin H., Ding H. (2011). Predicting ion channels and their types by the dipeptide mode of pseudo amino acid composition. J. Theor. Biol..

[37] Chou K.C. (2001). Prediction of protein cellular attributes using pseudo-amino acid composition. Proteins.

[38] Nieto J.J., Torres A., Georgiou D.N., Karakasidis T.E. (2006). Fuzzy polynucleotide spaces and metrics. Bull. Math. Biol..

[39] Georgiou D.N., Karakasidis T.E., Nieto J.J., Torres A. (2010). A study of entropy/clarity of genetic sequences using metric spaces and fuzzy sets. J. Theor. Biol..

[40] Zhao X., Li X., Ma Z., Yin M. (2011). Prediction of lysine ubiquitylation with ensemble classifier and feature selection. Int. J. Mol. Sci..

[41] Liu B., Xu J., Zou Q., Xu R., Wang X., Chen Q. (2014). Using distances between Top-n-gram and residue pairs for protein remote homology detection. BMC Bioinform..

[42] Magnan C.N., Randall A., Baldi P. (2009). SOLpro: Accurate sequence-based prediction of protein solubility. Bioinformatics.

[43] John A.C., Mona S. (2007). Predicting functionally important residues from sequence conservation. Bioinformatics.

[44] Schaffer A.A., Aravind L., Madden T.L., Shavirin S., Spouge J.L., Wolf Y.I., Koonin E.V., Altschul S.F. (2001). Improving the accuracy of PSI-BLAST protein database searches with composition-based statistics and other refinements. Nucleic Acids Res..

[45] Tao P., Liu T., Li X., Chen L. (2015). Prediction of protein structural class using tri-gram probabilities of position-specific scoring matrix and recursive feature elimination. Amino Acids.

[46] Zhang L., Zhang C., Gao R., Yang R. (2015). An ensemble method to distinguish bacteriophage virion from non-virion proteins based on protein sequence characteristics. Int. J. Mol. Sci..

[47] Zuo Y.C., Peng Y., Liu L., Chen W., Yang L., Fan G.L. (2014). Predicting peroxidase subcellular location by hybridizing different 4 descriptors of Chou’s pseudo amino acid patterns. Anal. Biochem..

[48] Eichner J., Topf F., Drager A., Wrzodek C., Wanke D., Zell A. (2013). TFpredict and SABINE: Sequence-based prediction of structural and functional characteristics of transcription factors. PLoS ONE.

[49] Zhang J., Zhao X., Sun P., Ma Z. (2014). PSNO: Predicting cysteine s-nitrosylation sites by incorporating various sequence-derived features into the general form of Chou’s PseAAC. Int. J. Mol. Sci..

[50] Hayat M., Tahir M., Khan S. (2014). Prediction of protein structure classes using hybrid space of multi-profile Bayes and bi-gram probability feature spaces. J. Theor. Biol..

[51] Sharma A., Lyonsm J., Dehzangi A., Paliwal K.K. (2013). A feature extraction technique using bi-gram probabilities of position specific scoring matrix for protein fold recognition. J. Biomol. Struct. Dyn..

[52] Ramoser H., Muller-Gerking J., Pfurtscheller G. (2000). Optimal spatial filtering of single trial EEG during imagined hand movement. IEEE Trans. Rehabil. Eng..

[53] Chawla N.V., Bowyer K.W., Hall L.O., Kegelmeyer W.P. (2002). SMOTE: Synthetic minority over-sampling technique. J. Artif. Intell. Res..

[54] Ding C., Yuan L.F., Guo S.H., Lin H., Chen W. (2012). Identification of mycobacterial membrane proteins and their types using over-represented tripeptide compositions. J. Proteom..

[55] Ebina T., Suzuki R., Tsuji R., Kuroda Y. (2014). H-DROP: An SVM based helical domain linker predictor trained with features optimized by combining random forest and stepwise selection. J. Comput. Aided Mol. Des..

[56] Ebina T., Toh H., Kuroda Y. (2011). DROP: An SVM domain linker predictor trained with optimal features selected by random forest. Bioinformatics.

[57] Breiman L. (2001). Random forests. Mach. Learn..

[58] Kandaswamy K.K., Pugalenthi G., Hartmann E., Kalies K.U., Moller S., Suganthan P.N., Martinetz T. (2010). SPRED: A machine learning approach for the identification of classical and non-classical secretory proteins in mammalian genomes. Biochem. Biophys. Res. Commun..

[59] Mohamed T.P., Carbonell J.G., Ganapathiraju M.K. (2010). Active learning for human protein-protein interaction prediction. BMC Bioinform..

[60] Witten I.H., Frank E. (2005). Data Mining: Practical Machine Learning Tools and Techniques.

[61] Chou K.C., Zhang C.T. (1995). Prediction of protein structural classes. Crit. Rev. Biochem. Mol. Biol..

[62] Fawcett T. (2006). An introduction to ROC analysis. Pattern Recognit. Lett..

